# Molmil: a molecular viewer for the PDB and beyond

**DOI:** 10.1186/s13321-016-0155-1

**Published:** 2016-08-26

**Authors:** Gert-Jan Bekker, Haruki Nakamura, Akira R. Kinjo

**Affiliations:** 1 Laboratory of Protein Informatics, Institute for Protein Research, Osaka University, 3-2 Yamadaoka, Suita, Osaka 565-0871 Japan; 2 Graduate School of Frontier Biosciences, Osaka University, Suita, Osaka 565-0871 Japan

**Keywords:** WebGL, Protein structure visualization, Molecular dynamics

## Abstract

We have developed a new platform-independent web-based molecular viewer using JavaScript and WebGL. The molecular viewer, Molmil, has been integrated into several services offered by Protein Data Bank Japan and can be easily extended with new functionality by third party developers. Furthermore, the viewer can be used to load files in various formats from the user’s local hard drive without uploading the data to a server. Molmil is available for all platforms supporting WebGL (e.g. Windows, Linux, iOS, Android) from http://gjbekker.github.io/molmil/. The source code is available at http://github.com/gjbekker/molmil under the LGPLv3 licence.

**Electronic supplementary material:**

The online version of this article (doi:10.1186/s13321-016-0155-1) contains supplementary material, which is available to authorized users.

## Background

Molecular viewers are a vital tool for our understanding of protein structures and functions. The shift from regular desktop platforms such as Windows, Mac OSX and Linux, to mobile platforms such as iOS and Android in the last half-decade, however, prevents traditional online molecular viewers such as PDBj’s previously developed jV [1] and the popular Jmol [2] from running on these new platforms as these platforms do not support Java Applets. For mobile platforms a native application (i.e. an application specifically designed and optimized for each of these platforms) can be created and distributed via their respective application stores. However, with new platforms on the horizon, or already available, in addition to the already established desktop platforms, it would be a tedious and inefficient job to make a molecular viewer available on all platforms, current and future.

In the same half-decade however, web browsers have also made significant advances, particularly with their JavaScript engines. Further advances in the form of CSS3 [3] and HTML5 [4] have made the web an interesting platform for developers. Mobile platforms also offer these advanced web browsers, while some newer mobile platforms such as Mozilla’s Firefox OS [5] and Ubuntu Mobile [6] are oriented around HTML5 applications. Since these advanced web browsers are available on a wide range of platforms, it seems to be the perfect platform to develop a molecular viewer for.

One of the earliest attempts to switch to a Java independent molecular web viewer was done by the developers of the popular molecular viewer Jmol who developed an alternate molecular viewer named JSmol [7] to run directly within the web browser. They simply used a program known as java2script to convert Jmol’s Java code into JavaScript. JSmol however runs noticeably slower compared to Jmol for relatively small protein structures and quickly becomes unusable for larger structures. Desktop applications such as Chimera [8], VMD [9], PyMol [10] and Yasara [11] make use of GPU based hardware acceleration using OpenGL [12]. By making use of OpenGL, one can leverage the immense power of GPUs to accelerate these molecular viewers. GPUs have traditionally been designed for this purpose; i.e. to accelerate the generation of 3D images, but more recently GPUs have also been re-purposed for general purpose calculations in e.g. Molecular Dynamics [13, 14]. So wouldn’t it be great if this power were somehow also available inside web browsers?

Enter WebGL. WebGL was developed by Khronos [15] to allow JavaScript applications running in the web browser to take advantage of OpenGL ES 2.0 [16] compatible GPUs which had been specifically designed for mobile devices. While WebGL has been available for several years in Chrome [17] and Firefox [18], WebGL support was only recently added to Microsoft’s Internet Explorer and Apple’s Safari, including iOS. We have developed a new web based molecular viewer, Molmil, which can take advantage of GPU hardware acceleration using WebGL. It runs on a wide range of platforms such as Windows, Linux, Mac OSX, Android and iOS. Finally, we have embedded Molmil within various services offered by Protein Data Bank Japan [1, 19–21] to demonstrate its capabilities.

## Implementation and capabilities

Molmil has been designed as a light-weight and full-featured viewer for the PDB. As such, Molmil can load legacy PDB flat files, PDBx/mmCIF [22] and PDBML [23] formatted files. Molmil can also load a custom format which we call PDBx/mmJSON, which is a JSON [24] version of the PDBx/mmCIF data. Other formats which Molmil supports are GRO, MOL2, MDL, CCP4 [25] (for electron density maps and EM data), MyPresto’s trajectory format [14, 26], Gromacs’ TRR and XTC trajectory formats [13] and our own developed MPBF polygon format which we are using for our eF-site service [1] for large structures. Users can also load these files from their local hard drive.

The PDBx/mmJSON (mmJSON for short) format was specifically developed for Molmil. In a nutshell, it is a JSON representation of the mmCIF format, made by translating the data structure based on the STAR syntax [27], into a series of associative arrays, standard arrays, integers, floats and null values. A major advantage of this format is that it can be directly interpreted by modern browsers, that is, a typed data structure is available once the JSON data has been parsed by a browser. Such is not the case for PDBML (XML) or mmCIF formats. In addition, the file size is generally smaller in the mmJSON format than in PDBML or mmCIF formats. An analysis showed that the compressed mmJSON is on average approximately 33 or 56 % smaller than a compressed mmCIF or PDBML formatted file, respectively, making it more suitable for web deployment. For large structures another derivative of mmJSON is currently under development to only include the minimal amount of information in the file required to display the backbone structure. This mmJSON variant is not yet offered as a service by PDBj, but can be previewed by using Molmil: http://gjbekker.github.io/molmil/#molmil.loadPDBlite(‘3j3q’). Implementation details regarding the parsers used to load the mmCIF data, as well as information regarding the mmJSON format and its relation with the mmCIF format is given on its Github page at http://github.com/gjbekker/cif-parsers.

### Displaying large entries

**Fig. 1:**
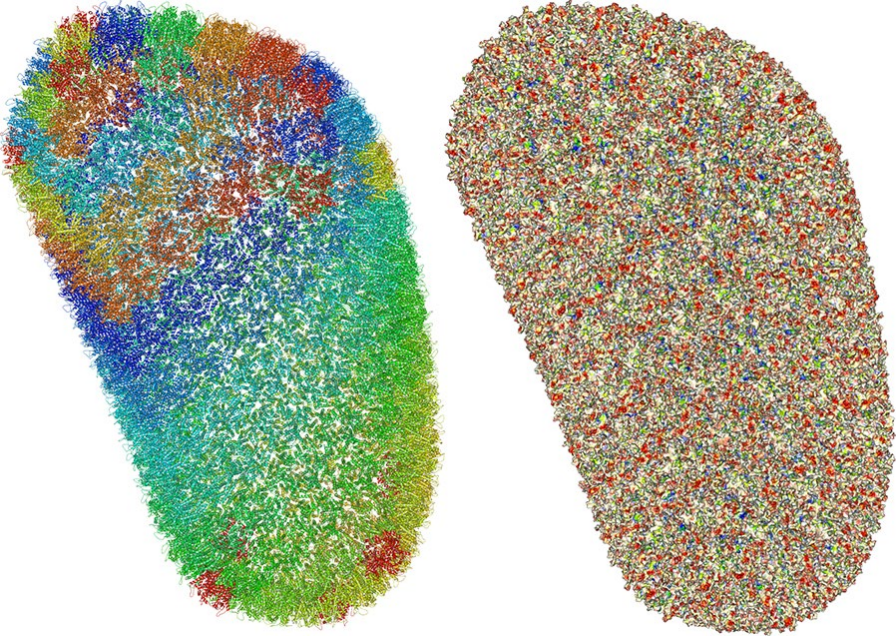
Rendering of 3j3q using Molmil. 3j3q [28] is the largest PDB entry to date (with more than 2.4 million atoms) and can be visualized using Molmil. *Left*: cartoon rendering with group colouring. *Right*: rendering of the molecular surface, coloured by electrostatic potential and hydrophobicity generated by eF-site [1]

One of the design goals of Molmil was to create a molecular viewer which can produce high quality images suitable for publications. On the other hand, it should also be able to scale to very large structures available in the Protein Data Bank (PDB) consisting of hundreds of thousands to millions of atoms. By default WebGL can only display a limited amount of polygons. However, by using an extension to WebGL (OES_element_index_uint) which is available on all modern platforms, it becomes possible to efficiently render very large or highly detailed structures. Molmil uses this extension to build high quality geometry for small and medium sized structures and dynamically scale down the quality as the size of the structure increases to gigantic proportions (such as the HIV-1 capsid; 3j3q [28], see Fig. 1). Note that to be able to load these gigantic structures, an adequate amount of memory is still required, which is often not available on smartphones, tablets and older systems. To enable high quality lighting even when using polygon models of low detail for these gigantic structures, Molmil uses tuned Phong shading [29] which can accurately calculate the lighting even for simplistic polygon models.

### Rendering and colouring modes

**Fig. 2:**
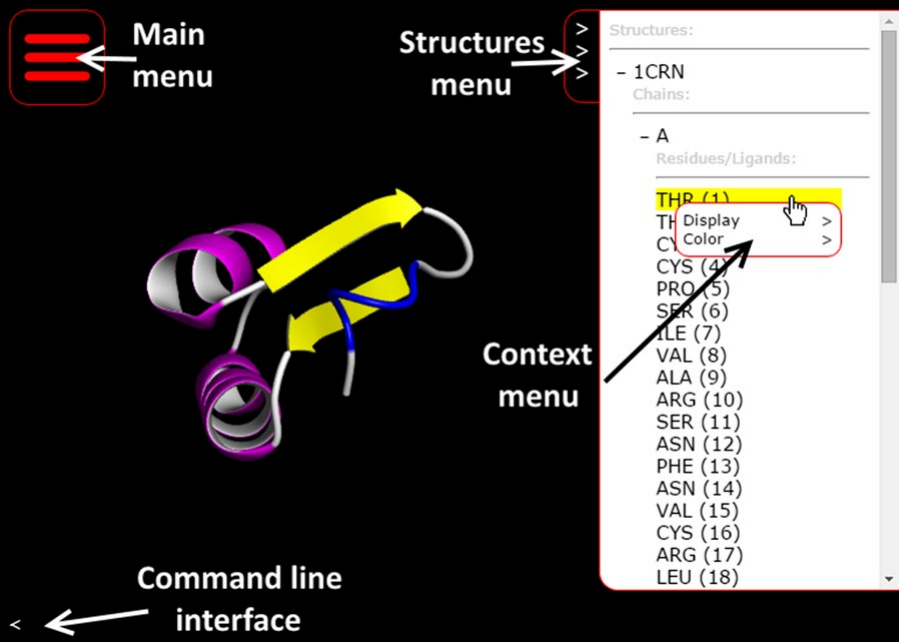
Screenshot of Molmil loading the url: http://gjbekker.github.io/molmil/#molmil.loadPDB(‘1crn’). Indicated are the Main menu *on the left* which can be used to load files, save PNG images, modify settings and play animations if available. The structures menu *on the right* can be shown by clicking on the indicated button. The context menu which can be displayed by right clicking on any structures, chains or residues/ligands listed within the structures menu. The command line interface which can be shown by clicking on the indicated button

Molmil supports several rendering and colouring modes which can be selected using a point-and-click based menu. In the top-right corner of Molmil’s canvas the structures menu can be enabled (see also Fig. 2). This structures menu lists all the loaded structures in the current canvas. Via a hierarchical tree selection system chains and residues belonging to the structure can be explored. Right clicking on the structures, chains or residues displays a context menu via which users can change the display and colour modes. Double clicking on the residues causes Molmil to focus and jump to the selected residue. Furthermore, right-clicking on the atoms or cartoon rendering within the canvas also enables users to interactively modify the rendering and colour mode of the selected atom or the residue/chain the atom belongs to. These controls are reminiscent from Yasara [11], which uses a similar hierarchical structures menu and context menu to modify the display and colour modes of the loaded structures. Molmil currently supports VDW, ball-and-stick, stick, Cα-trace, tube, cartoon, rocket and coarse surface representations. Structures can be coloured by their secondary structure elements, CPK, group (blue-to-red gradient), chain, ABEGO [30] or custom colour assignments. Views can be outputted as high quality PNG images, suitable for publication without requiring additional plugins. An overview of Molmil’s various rendering modes can be found in Additional file 1: Appendix I.

### Playing animations

It is also possible to load PDB files with multiple models and play them as animations, by using the “Animation” control panel which is accessible from Molmil’s main menu (see Fig. 2). PDBj’s Promode Elastic service [20] uses this functionality. It is also possible to use this animation functionality using alternative formats to e.g. play Molecular Dynamics trajectories. Currently myPresto’s [26] trajectory format and Gromacs’s [13] TRR and XTC trajectory formats are supported by Molmil and support for other formats can be easily added. Although web browsers can be used to produce PNG images by Molmil, they cannot make MP4 movies. However, by using a server-side REST service it is possible to stitch the images of each frame in the trajectory together to build a movie. For obvious reasons (potential abuse) we have not made this functionality available as a public REST service, but users can obtain this application from Molmil’s Github page which they can then run on their own workstation so that their own machine will then build the movie. Using this tool, Molmil can e.g. be used to produce high quality MP4 videos of molecular simulation trajectories.

### Command-line interface

Molmil also has an embedded command line which can be used to manually perform actions. The command line is capable of executing arbitrary JavaScript code and has access to Molmil’s application program interface (API), which can also be used by third party developers to embed and extend Molmil within their own web page/app. The command line can also be used to perform simple tasks such as selecting chains, residues or atoms and changing the display and colouring of them. Third party developers can also add support for their own custom file formats, or add additional functionality. The embedded viewers on the PDBj website have been implemented in this manner. Details and examples of the Molmil API are described on Molmil’s Github page http://github.com/gjbekker/molmil/wiki. The command line can be accessed via the “<” symbol in the lower left corner of the canvas (see also Fig. 2). Finally, the commands can also be embedded within the URL for easy sharing via email, web pages and social media (see Fig. 2 for a simple example URL).

### Molmil availability

Molmil has seen several major revisions since its initial release in December 2013. Molmil Beta featured a prototype WebGL viewer which was implemented directly into PDBj’s web application. Molmil 0.9 was released in September 2014 and had all the basic functionalities a molecular viewer needs such as reading various formats and supporting multiple display and colour modes via a point-and-click based menu. The latest major version of Molmil, Molmil 1.0, was released in October 2015 which focused on optimizations and extending the feature set of Molmil and also added a powerful JavaScript based command line interface.

Molmil has been integrated in many of PDBj’s services [21], including: Mine PDB Explorer Asymmetric Unit viewer; Mine PDB Explorer Biological Unit viewer; EDMap viewer (for electron densities); Sequence Navigator; Structure Navigator; Promode Elastic (for viewing protein mobility analysed via Normal Mode Analysis) and Chemie (PDBj’s new chemical component service). More recently, the eF-site [1] service (for viewing the electrostatic surface of proteins) was also updated with an integrated Molmil viewer and the Biological Unit viewer was also upgraded. Furthermore, high quality PNG images of all the released PDB entries on the PDBj website have also been generated by Molmil in an automated manner. More information regarding Molmil as well as use-cases and command-line examples can be found at http://github.com/gjbekker/molmil/wiki.

## Discussion

Molmil requires the browser to support WebGL. Furthermore, for optimal use, the OES_element_index_uint must also be supported. When loading very large structures such as 3j3q, there are additional requirements to the user’s software and hardware. Since Molmil requires at least 6 GB of memory to load the structure into memory, the user’s device must be equipped with 8 GB of memory. Furthermore, to enable Molmil to make use of this large amount of memory, a 64-bit browser and operating system are required. Finally, due to limitations of Chrome’s JavaScript engine, even Chrome’s 64-bits version is incapable of loading 3j3q. Mozilla’s Firefox and Apple’s Safari however have no such problem with the 64-bit versions. Note that it will still take tens of seconds to several minutes depending on the web browser, the user’s hardware such as the processor and the user’s internet connection since 3j3q’s compressed data file is still about 40 MB large. The implementation at PDBj’s web application will automatically detect the user’s software and hardware environment and will display a link to the entry’s embedded Molmil page if the entry is compatible with the user’s system. All regular entries and most large structure entries however have no problems loading on low-end devices, as long as WebGL is supported.

Although Molmil exposes some of PyMol’s commands via Molmil’s command line, support is currently limited to a small subset of commands for selection, styling and colouring of structures. Also, although Molmil can be used to load CCP4 files for electron densities and generate coarse surfaces for proteins, high quality surfaces such as MSMS and solvent-accessible surfaces are currently not yet supported, partly because of the high computational requirements involved in generating highly detailed surfaces.

Since Molmil’s initial release in 2013, other WebGL-based molecular viewers have also emerged. E.g. JSmol (JSmol/WebGL) has been extended with a basic WebGL based viewer and PV [31] was developed by SWISS-MODEL and is currently also being used by the RCSB-PDB. Other WebGL-based molecular viewers are listed in Additional file 1: Appendix II. Molmil however offers high quality graphics due to using highly detailed polygon models when possible, while it can scale up to display very large structures by dynamically reducing the quality to render these large structures in combination with a finely tuned Phong shader for smooth and realistic shading even for lower detailed models. Furthermore, Molmil can also be used to easily load files from the users’ hard drive, including molecular simulation trajectories, and comes with a readily accessible command line interface, which is also programmable by embedding the commands in the URL, as shown in e.g. Fig. 2.

## Conclusions

We have designed a versatile, high performance, high quality molecular viewer for the web. We have also deployed the molecular viewer on the PDBj web application as part of various services to aid users as well as demonstrate some of the capabilities of Molmil. The source code is available on GitHub as well as documentation on how to use and/or deploy Molmil on your own website. Questions or requests can be made either via Molmil’s GitHub page or via our contact page http://pdbj.org/contact.

## Availability and requirements

Project name: Molmil; Project home page: http://github.com/gjbekker/molmil; Operating system(s): Platform independent; Programming language: JavaScript, CSS3, HTML5, WebGL, GLSL; Other requirements: WebGL; Licence: GNU LGPLv3; Restrictions for use by non-academics: None.

## Additional file

10.1186/s13321-016-0155-1 Appendices; Example renderings using Molmil & Overview of alternative WebGL based molecular viewers.

## Abbreviations

PDB: Protein Data Bank
PDBj: Protein Data Bank Japan
CSS3: Cascade Style Sheets version 3
HTML5: hyper text markup language version 5
GPU: graphics processing unit
REST: representational state transfer
EM: electron microscopy
MPBF: molmil polygon binary format
API: application programming interface
VDW: Van Der Waals
PNG: portable network graphics
XML: extendible meta language
