# Supplementary material for: Molmil: a molecular viewer for the PDB and beyond
Source: J Cheminform. 2016 Aug 26;8(1):42. doi: 10.1186/s13321-016-0155-1 (PMC5002144; doi:10.1186/s13321-016-0155-1)
Supplement: Supplementary file 1 — 10.1186/s13321-016-0155-1 Appendices; Example renderings using Molmil & Overview of alternative WebGL based molecular viewers. [file 13321_2016_155_MOESM1_ESM.pdf]

APPENDIX I: Example renderings using Molmil

Cartoon rendering with group colouring of 1crn:<br>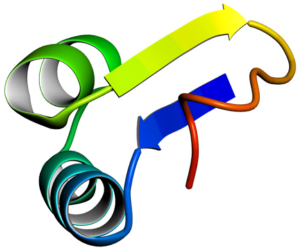	Cα trace rendering with group colouring of 1crn:<br>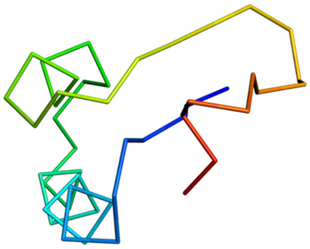
Wireframe rendering with ABEGO (30) colouring of 1crn:<br>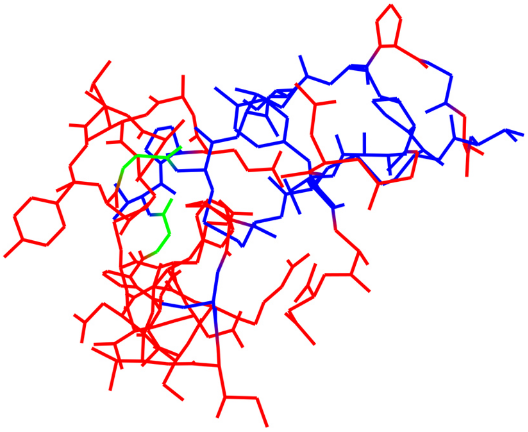	CPK rendering with element colouring of 1crn:<br>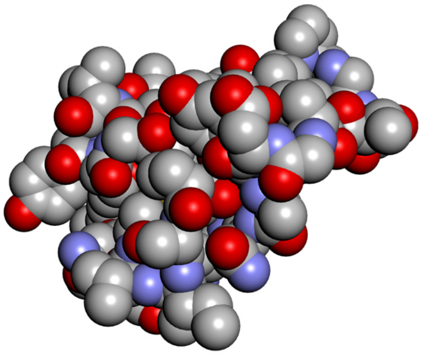

Cartoon rendering with ABEGO (30) colouring of 1crn:<br>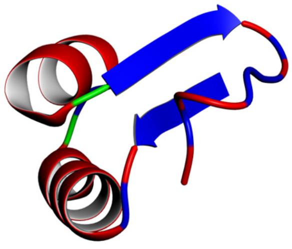	Stick rendering with element colouring of 1crn:<br>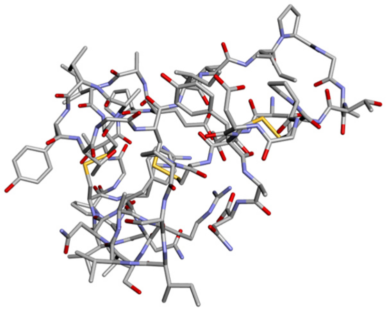
Ball-and-stick rendering with element colouring of 1crn:<br>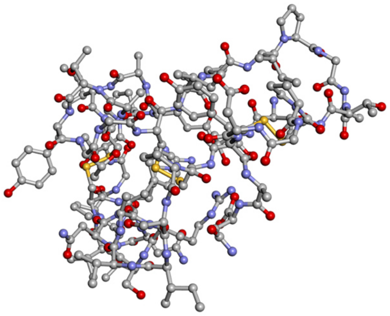	Cartoon rendering with side-chains as sticks with secondary structure and element colouring of 1crn:<br>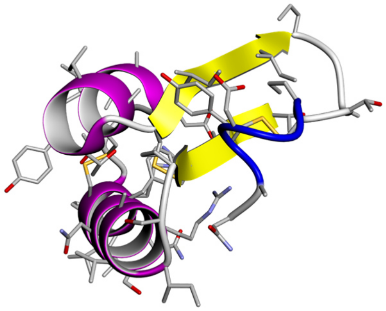

Tube rendering with secondary structure colouring of 1crn: 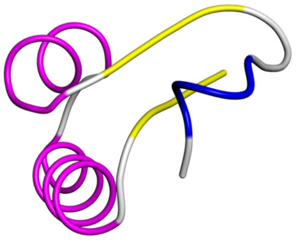	Rocket rendering with group coloring of 2prg: 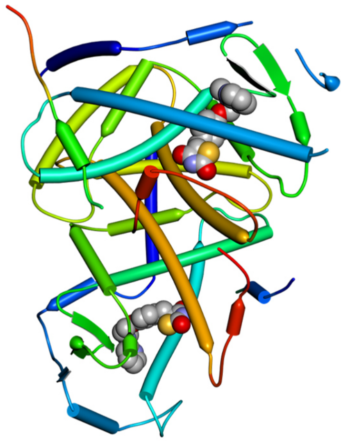
Coarse surface rendering with chain colouring of 2prg: 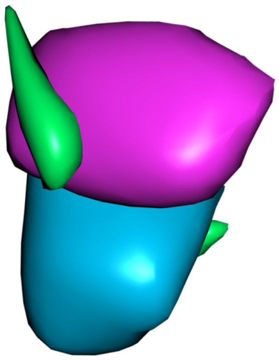	Rocket rendering (simplified) with secondary structure colouring of 3j3q: 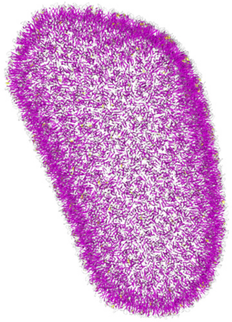

Rocket rendering (simlified) with secondary structure colouring of 3j3q (simplified) and fog to improve depth perception:<br>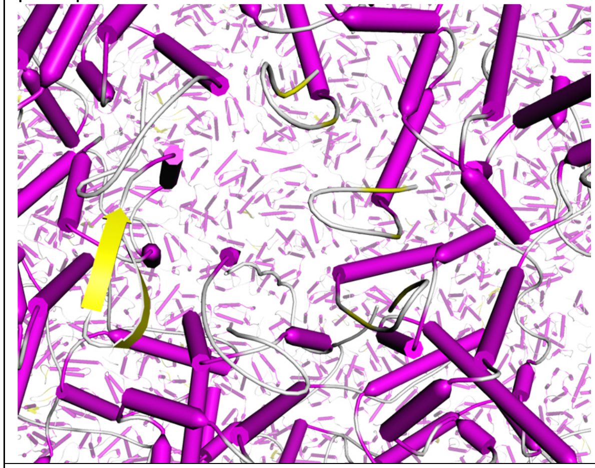	Coarse surface rendering with chain colouring of 3j3q (simplified):<br>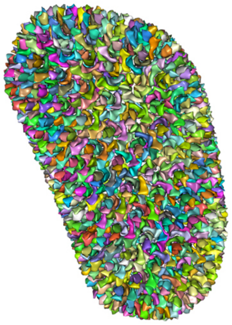
Cartoon and coarse surface rendering of the biological unit of 1bbt with group and chain colouring for the assymetric chains and the remaining chains respectively:<br>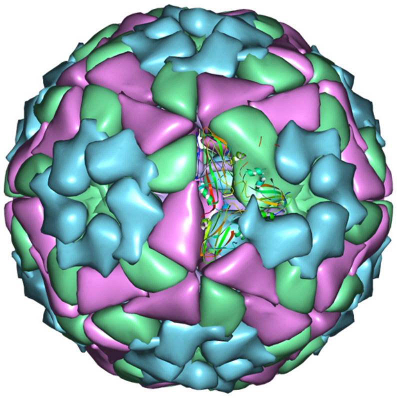	Cartoon rendering of the biological unit of 1bbt with group and chain colouring for the assymetric chains and the remaining chains respectively:<br>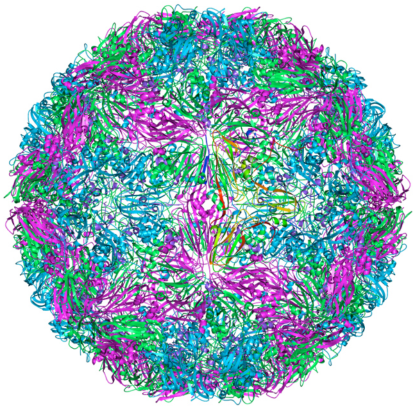

<table>
<tr>
<td>Cartoon and wireframe rendering of the biological unit of 1bbt with group and chain colouring for the assymetric chains and the remaining chains respectively:
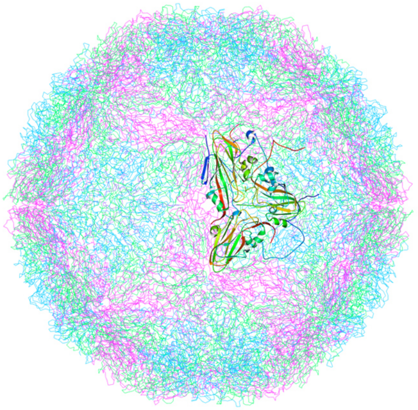</td>
<td>Stick rendering and iso-surface of the electron density of 1ubq, part of PDBj's EDMap service:
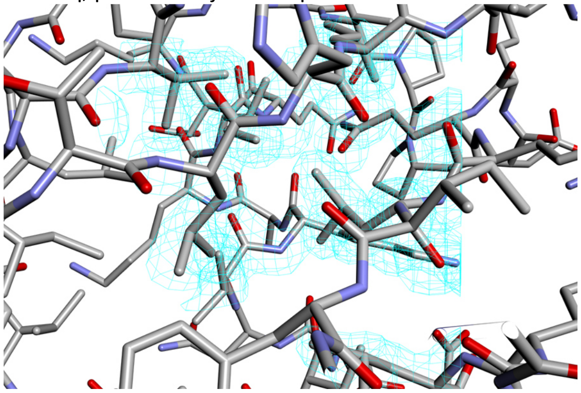</td>
</tr>
<tr>
<td>Iso-surface rendering of 1ubq with the surface colored by electrostatic potential and hydrophobicity, part of PDBj's eF-site service:
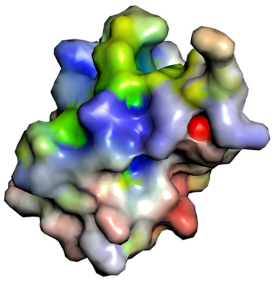</td>
<td>Cartoon rendering of 1ubq with secondary structure colouring and displacement vectors, part of PDBj's Promode Elastic service:
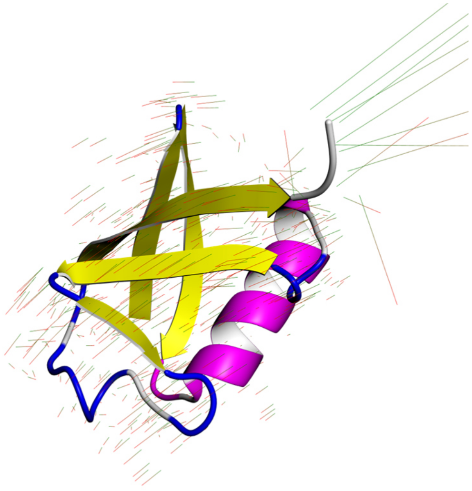</td>
</tr>
</table>

APPENDIX II: Overview of alternative WebGL based molecular viewers

GLmol	Basic PDB viewer, one if not the earliest WebGL viewer<br>http://webglmol.osdn.jp/index-en.html
JSmol/WebGL (31)	Limited functionality version of Jmol using HTML5+WebGL<br>http://jmol.sourceforge.net/
PV (30)	PDB viewer developed by Swiss-Model and used by the RCSB<br>https://github.com/biasmv/pv
3Dmol.js (32)	Developed by the university of Pittsburg.<br>https://github.com/3dmol/3Dmol.js
NGL (33)	Developed by the Charité–Universitätsmedizin Berlin<br>https://github.com/arose/ngl
ChemDoodle WC (34)	Commercial library (free to use) developed by iChemLabs<br>https://web.chemdoodle.com/
Speck	High quality renderer for small molecules<br>https://github.com/wwwtyro/speck
iCn3D	PDB viewer used by the NCBI<br>https://github.com/ncbi/icn3d
